# The use of a hybrid Sequential Biofiltration System for the improvement of nutrient removal and PCB control in municipal wastewater

**DOI:** 10.1038/s41598-017-05555-y

**Published:** 2017-07-14

**Authors:** Edyta Kiedrzyńska, Magdalena Urbaniak, Marcin Kiedrzyński, Adam Jóźwik, Agnieszka Bednarek, Ilona Gągała, Maciej Zalewski

**Affiliations:** 1 European Regional Centre for Ecohydrology of the Polish Academy of Sciences, ul. Tylna 3, 90-364 Lodz, Poland; 2 Department of Applied Ecology, Faculty of Biology and Environmental Protection, University of Lodz, ul. Banacha 12/16, 90-237 Lodz, Poland; 3 Department of Geobotany and Plant Ecology, Faculty of Biology and Environmental Protection, University of Lodz, ul. Banacha 12/16, 90-237 Lodz, Poland; 4 Department of Informatics, Faculty of Physics and Applied Informatics, University of Lodz, Pomorska 149/153, 90-236 Lodz, Poland; 5 Department for Mathematical Modelling of Physiological Processes, Institute of Biocybernetics and Biomedical Engineering, Polish Academy of Sciences, Trojdena 4, 02-109 Warsaw, Poland

## Abstract

This article aims to evaluate the efficiency of an innovative hybrid Sequential Biofiltration System (SBS) for removing phosphorus and nitrogen and polychlorinated biphenyls (PCBs) from original municipal wastewater produced by a Wastewater Treatment Plant under authentic operating conditions. The hybrid SBS was constructed with two barriers, a geochemical (filtration beds with limestone, coal and sawdust) and a biological barrier (wetlands with *Glyceria*, *Acorus*, *Typha*, *Phragmites*), operating in parallel. Significant differences were found between inflow and outflow from the SBS with regard to wastewater contaminant concentrations, the efficiency of removal being 16% (max. 93%) for Total Phosphorus (TP), 25% (max. 93%) for Soluble Reactive Phosphorus (SRP), 15% (max. 97%) for Total Nitrogen (TN), 17% (max. 98%) for NO_3_^–^N, and 21% for PCB equivalency (PCB EQ). In the case of PCB EQ concentration, the highest efficiency of 43% was obtained using beds with macrophytes. The SBS removed a significant load of TP (0.415 kg), TN (3.136 kg), and PCB EQ (0.223 g) per square meter per year. The use of low-cost hybrid SBSs as a post-treatment step for wastewater treatment was found to be an effective ecohydrological biotechnology that may be used for reducing point source pollution and improving water quality.

## Introduction

The combination of anthropogenic activities in the drainage area and intensified export of nutrients to the water environment from point and diffuse sources have led to over-enrichment, which is strongly observed worldwide^1–3^. The municipal wastewater discharged from Wastewater Treatment Plants (WWTPs) often represents a significant source of phosphorus (P) and nitrogen (N) load^3–6^ and polychlorinated biphenyls (PCBs)^7, 8^, which are discharged into rivers, reservoirs or coastal zones. Nutrient loads promote eutrophication and abnormal phytoplankton growth; they also encourage the occurrence of toxic cyanobacteria blooms, resulting in the degradation of water quality and threatening the health of humans and animals. PCBs, in turn, are classified as Persistent Organic Pollutants, and comprise a group of substances of toxic, persistent and bioaccumulative properties. According to the U.S. Environmental Protection Agency, PCBs have been shown to cause cancer in humans and animals, and also influence the immune, endocrine, neurological and reproductive systems, posing a serious risk for living organisms and the well-being of the ecosystem^9, 10^. Therefore, to prevent eutrophication and degradation of water bodies, and safeguard human health, the nutrient and PCB loads flowing into the surrounding environment need to be reduced. Legislation on the pollutant content of municipal WWTP outflow is becoming stricter in European Union (Urban Waste Water Treatment Directive 91/271/EEC), especially regarding nutrients that are also strictly controlled at the national level. Nevertheless, the legislation criteria for organic compounds such as PCBs, which may be introduced into water bodies via their presence in treated wastewater, are still not sufficient to properly monitor and protect aquatic ecosystems. With regard to the allowable discharge limits of PCBs given in the EU directives, only EQS Directive (2013/39/EC), which established the list of new priority substances and priority hazardous substances in water policy and introduced an obligation to monitor their concentrations in water ecosystems, included dioxin-like compounds such as PCBs. However, although PCBs have been identified as priority hazardous substances, no allowable concentration in inland surface waters has been provided. Similarly, the Urban Wastewater Directive (91/271/EEC) has no information concerning the allowable limits of PCBs in wastewater discharged into the surface water. Hence, at the EU level, these compounds are not monitored in inland water nor in wastewater. Such limits exist in national (Polish) law regulations: for example, one of the most important Polish regulations in the field of water policy, the Water Law (OJ 2001 No. 115, item 1229, act of July 19 2001, the Water Law), prohibits the discharge of indicator PCBs (PCB 28, 52, 101, 138, 153 and 180) into river ecosystems through WWTP outlets. However, no such limit exists with respect to other congeners of PCBs, including the toxic ones that have the greatest influence on the quality status of water bodies. Another set of Polish regulations, the Ministry of the Environment Regulation dated 24 July 2006 on the conditions to be met when discharging sewage into waters or soil, and on substances of particular adverse impact on the water environment (Journal of Laws 2006 no. 137, item 984), also prohibits the discharge of PCBs with treated wastewater, but this regulation applies only to industrial WWTPs. Consequently, municipal WWTPs are not monitored in terms of PCB discharges into the water bodies. This is a vital problem, as the majority of municipal WWTPs are not effective at PCB removal^11–17^. This is also the case in Poland: it has been demonstrated that all WWTPs monitored in a previous study in central Poland were unable to completely remove PCBs and other toxic organic compounds from wastewater during conventional purification processes^7, 8^. As a consequence, PCBs are detected not only in treated wastewater but also in recipient inland water, creating a risk for aquatic organisms.

On the other hand, the ratification of the Water Framework Directive (2000/60/EC) requires that all member states put measures into place to achieve ‘good chemical and biological status’ in controlled waters by the year 2015, through the promotion of sustainable management techniques. This piece of legislation requires all liquid waste produced by industry to be treated, and comply with specified standards before discharge into a water body. These European Directives encourage the development of alternative wastewater treatment techniques that meet legislation criteria whilst also providing a more environmentally viable solution. Accordingly, we as a society need to adopt more innovative and comprehensive solutions, such as those offered by ecohydrological biotechnology. One such example is the hybrid Sequential Biofiltration System (SBS), which has been found to be a relatively simple and inexpensive way to effectively remove biogenic compounds and PCBs from wastewater. Ecohydrology provides a scientific understanding of the interplay between hydrology and biota in an environment, and a systemic framework on how to use ecosystem processes for water quality improvement^18, 19^. Reducing anthropogenic pollution from WWTPs by implementing solutions based on the ecohydrological biotechnologies concept, such as closing nutrient cycles in the catchment^3^ and enhancing the retention of nutrients in the terrestrial ecosystem, could increase the sustainability of surface and coastal water bodies^2^. Methods based on Ecohydrology tend to be less expensive, more environmentally friendly and socially acceptable. Therefore, it is necessary to harmonise hydrotechnical solutions with ecohydrology^20^ and ecological engineering^21^ and environmental biotechnologies in river catchments. Taking this synergetic approach on the particular catchments will most probably result in significant reductions of nutrient and PCB loads on aquatic systems.

**Figure 1:**
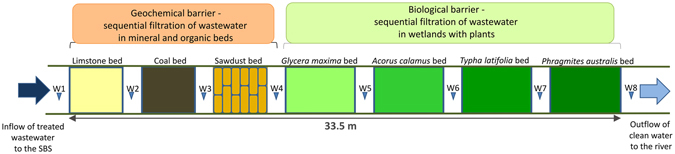
The design of a hybrid Sequential Biofiltration System (SBS) for the purification of wastewater effluent from a small WWTP, central Poland.

One such alternative is represented by the use of biofiltration systems such as constructed wetlands and the hybrid Sequential Biofiltration System (Fig. 1); these promising soft-engineering techniques for the treatment of wastewater from various origins are considered to be efficient approaches to treating municipal and domestic wastewater^22^ and livestock wastewaters^23^, as well as effective means of intercepting agricultural^24^ and urban^25^ runoff. Constructed wetlands^26–28^, and natural floodplain river wetland^29–31^ have been evaluated as means of removing nutrients, PCBs^30^, arsenic and phenol^32^, as well as heavy metals^33^ from wastewater. The operations of these systems based on exploitation of the natural treatment processes involve complex interactions between soil, wastewater, plants, microorganisms and prevailing flow patterns^28^. In the current economic climate, low-cost solutions are very desirable. Biofiltration systems are two to three times more economically viable and labour efficient than current wastewater treatment options^34^, offer low construction and maintenance costs, and low energy consumption; they also have greater aesthetic value and create habitats for wildlife. Harrington and McInnes^23^ and O’Neill *et al*.^35^ report that no universal handbook on constructing constructed wetlands (CWs) exists: it is necessary to know the hydrological situation (discharge, velocity, flow, retention time), physical characteristics and chemical composition of the sewage needing treatment, as well as the characteristics of the drained landscape and the local climate. Therefore, the last ten years have seen a greater intensity of scientific investigation into the effectiveness of biofiltration systems in treating wastewater and removing contaminants. However, due to the growing pressure on water resources, there is an ever greater need to optimise the potential of these systems as a method of treating wastewater, and this demands a greater understanding of the treatment mechanisms. Therefore, to enable this water quality improvement, it is becoming increasingly important to develop an inexpensive and proficient, yet site-specific, wastewater treatment practice. Some international studies have demonstrated that the use of filtration zones with such substrates as slag, gravel or limestone are efficient methods for P and N removal from wastewater^36, 37^. However, most of these experiments were performed on the laboratory scale using synthetic P and N solutions^34^, which did not contain all the components (e.g., humic acids, organic colloids, and competing anions) occurring in real wastewater; this may affect the rate of P and N removal in a constructed wetland system^38, 39^. Significant differences in filter performance were observed when treating real effluent^38^. Additionally, few studies have been conducted on the removal of PCBs from real municipal wastewater in biofiltration systems^40^. Additionally, only a few experiments on sequentional constructed wetland systems have been conducted^41, 42^, and even fewer on the field scale using an existing municipal treatment plant^43^.

Very few, if any, previous studies at the time of writing have examined the use of a hybrid Sequential Biofiltration System (SBS) based on geochemical and biological barriers for the removal of pollutants from original municipal wastewater produced by a Wastewater Treatment Plant under authentic operating conditions. The present study, therefore, offers an innovative insight to the topic, and hence provides a unique set of findings which may act as a guideline for the construction of other wastewater biofiltration systems.

The aim of the study was to evaluate the efficiency of a system of seven beds that can be classified as a hybrid system: a geochemical barrier (three based on beds with mineral and organic materials: limestone, coal and sawdust) and a biological barrier (four beds with macrophytes species: *Glyceria maxima*, *Acorus calamus*, *Typha latifolia*, *Phragmites australis*) within a Sequential Biofiltration System, respectively (Fig. 1), to remove Total Suspended Solids (TSS), phosphorus and nitrogen compounds and PCBs from real municipal wastewater under authentic WWTP operating conditions.

## Results and Discussion

### Physico-chemical characteristics of sewage

**Figure 2:**
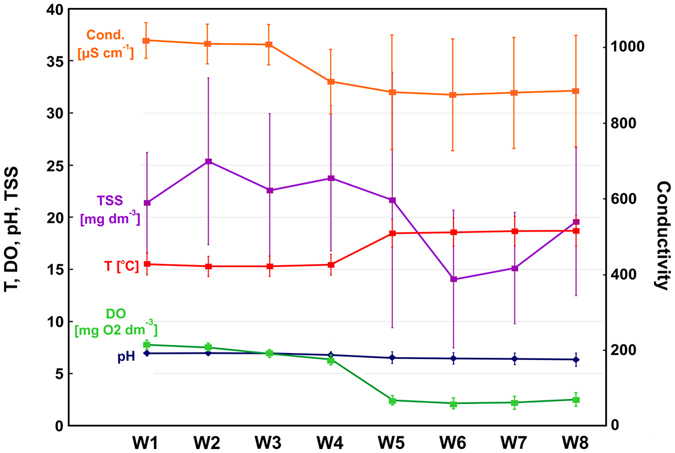
Physico-chemical characteristics of sewage at the outlet from the Wastewater Treatment Plant (W1) and sewage after flowing at particular zones of the Sequential Biofiltration System (W2–W8), central Poland. Thirteen samples were taken from each of eight stations, total number of analysed samples = 104. Whiskers include standard errors, and points represent the medians.

The physico-chemical characteristics of sewage collected from the SBS below the geochemical barrier with filtration beds (W2–W4) and below the biological barrier with macrophyte beds (W5–W8) were evaluated (Fig. 2). The mean discharge of wastewater from the WWTP in the study period was 79.1 m^3^ (max. 146 m^3^, min. 40 m^3^) per day. The mean temperature of wastewater outflowing from WWTP at station W1 and on the end section of the SBS at station W8 were 15.53 °C and 18.72 °C, respectively, while the respective pH values were 6.95 and 6.33 (Fig. 2). The concentration of dissolved oxygen (DO) fell from W1 (7.78 mg dm^−3^) to W8 (2.50 mg dm^−3^), indicating the process of denitrification that occurs in various beds along the SBS; this was especially intense in the four biologically active beds (W5–W8) with transplanted macrophytes (Figs 1 and 2). The physical parameters of the influent (W1), given as concentration of TSS (21.4 mg dm^−3^) and conductivity (1018 µS cm^−1^), indicated that the wastewater was highly polluted, and that this decreased along the SBS continuum to W8 (Fig. 2); this suggests that the treatment process of sewage flowing through the SBS system was an intense one. The high removal values observed for the mineral (10%) and organic (90%) forms of TSS (Fig. 2) may be accounted for by the settling, deposition and filtration of suspended matter. The organic compounds can be degraded both aerobically and anaerobically by heterotrophic microorganisms depending on the oxygen concentration in the bed ^30, 43, 44^.

### Effectiveness of phosphorus reduction

**Figure 3:**
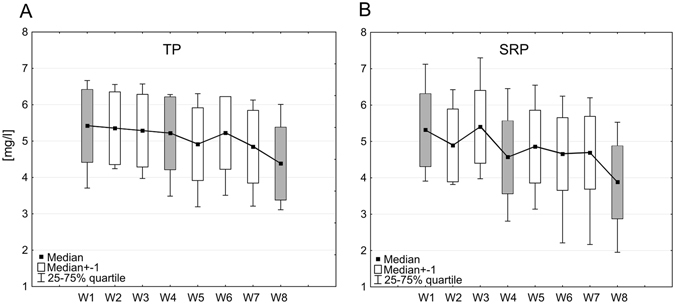
Concentrations of TP (**A**) and SRP (**B**) in wastewater outflowing from the WWTP (W1), then inflowing to the geochemical beds (W2–W4) and macrophyte beds (W5–W8) of the Sequential Biofiltration System, and then undergoing treatment. The shaded boxes represent the parameters of wastewater at: W1–inflow into the SBS, W4–below the geochemical barrier (below filtration beds), W8–below the plant barrier with macrophytes Thirty-six samples were taken from each of eight stations, total number of analysed samples = 288.

Biofiltration systems provide an environment where phosphorus occurs as phosphate in organic and inorganic compounds, and where all forms of phosphorus may subject to interconversion^35, 45^. The following physico-chemical transformation processes for phosphorous take place as wastewater flows though the system: adsorption, desorption, precipitation, dissolution, fragmentation, leaching, mineralization, sedimentation and burial. Of these, the major phosphorus removal processes in bofiltration systems are sorption, precipitation, sedimentation, and peat/soil accretion^43–45^. The key physico-chemical mechanisms of P removal from wastewater are known to act by the fixation of phosphate in the substrate by iron and aluminum, or allow P to become sorbed to wetland soils and sediments^38^. In addition, the very important process of phosphorous reduction is also biologically transformed by microbial and plant uptake ^43–45^. Generally, microorganisms are widely considered to represent the main force driving the wastewater treatment processes in biofiltration systems, as they can mineralize organic matter under both aerobic and anaerobic conditions^5, 46^. Plants constitute an indispensable component of biofiltration systems: although their effects on the treatment process vary according to the vegetation season, most studies have shown that systems with plants have higher removal efficiency of organics and nutrients than those without^47^. This is also confirmed by our present findings (Fig. 3). The key biological mechanism of P removal occurs in the dense plant root system in the rizosphere, where filtration and stimulated microbial activities increase phosphorus uptake and biomass production^5, 48^.

The efficiency of phosphorous reduction by the hybrid SBS was evaluated by measuring the TP and SRP concentrations in 36 samples of wastewater passing into (W1) and out from the system. The SBS itself consisted of three types of filtration beds, i.e. limstone (W2), coal (W3) and sawdust (W4), and four types of biologically-active bed planted with macrophytes: a *Glyceria maxima* bed (W5), *Acorus calamus* bed (W6), *Typha latifolia* bed (W7) and *Phragmites australis* bed (W8) (Fig. 3).

The results of the Friedman ANOVA test comparing the TP and SRP concentrations for all eight monitoring stations performed jointly is shown in Table 1. It can be observed that their concentrations depend on the stations, and decrease steadily from station W1 to W8 (from 5.13 to 4.31 mg TP dm^−3^), and (from 5.44 to 4.10 mg SRP dm^−3^, respectively) in the Sequential Biofiltration System. This decrease is statistically significant (p < 0.05) (Table 1). The mean percentage reduction between inflow (W1) and outflow (W8) was 16% for TP concentration (maximum 93%) and 25% for the SRP concentration (maximum 93%) (Table 1).

**Table 1:** A comparison of TP and SRP concentrations in wastewater considering all eight monitoring stations (W1–sewage outflowing from WWTP and W2–W8–sewage after cleaning in particular zones of the Sequential Biofiltration System).

Parameter	Station	Median	Mean concentration [mg dm^−3^]	N	S.D.
**TP**, ***p*** = **0**.000*	W1	5.42	5.13	36	1.93
	W2	5.35	5.26	36	2.13
	W3	5.29	5.11	36	1.82
	W4	5.21	4.99	36	2.04
	W5	4.91	4.71	36	1.91
	W6	5.23	4.83	36	1.91
	W7	4.85	4.73	36	2.12
	W8	4.38	4.31	36	2.03
**% Reduction of TP between W1–W8**		**Mean**	**16**		
		**Max**.	**93**		
		**Min**.	**−17**		
**SRP**, ***p*** = **0**.000*	W1	5.31	5.44	36	2.68
	W2	4.89	5.20	36	2.69
	W3	5.40	5.64	36	2.81
	W4	4.56	4.97	36	2.71
	W5	4..86	4.77	36	2.22
	W6	4.65	4.57	36	2.46
	W7	4.69	4.49	36	2.57
	W8	3.87	4.10	36	2.56
**% Reduction of SRP between W1–W8**		**Mean**	**25**		
		**Max**.	**93**		
		**Min**.	**−61**		

Friedman ANOVA test (*significance at p < 0.05). Percentage reduction of TP and SRP concentrations between stations W1 and W8 in the study period. A minus sign in % Reduction indicates increased concentration in outflowing wastewater. Thirty-six samples were taken from each of eight stations (total number of analysed samples = 288).

As a whole, the average removal efficiency of phosphate recorded in our study was higher than that presented by Jing *et al*.^49^, which ranged from 1.6% to 68.0%, and by Greenway and Woolley^50^ which was 13%. However, other studies, such as Brix and Arias^51^, carried out using vertical flow constructed wetland (VFCW), have reported 20 to 30% removal of phosphate, which is comparable to our obtained average values, i.e. 25% for SRP and 16% for TP. Abou-Elela and Hellal^43^ report the phosphate concentration in treated wastewater in a VFCW unit with a surface area of 457.56 m^2^ that was built close to a WWTP in North Cairo, Egypt, to range from 0.4 mg dm^−3^ and 2 mg dm^−3^, with an average percentage reduction of 62%. The high effectiveness of phosphorus reduction in this study may be attributed to the large area of the VFCW, the warmer climate and the use of plant species including *Canna*, *Cyprus papyrus* and *Phragmites australis* (similar to this study), which are known to be capable of high phosphorus uptake^43^.

A more detailed analysis is needed to identify the zone along the SBS system where the most effective reduction of TP and SRP concentrations were taking place. Therefore, pairs of variables of concentrations at particular stations were compared with the value at station W1 (inflow to the system) (Table 2). For this analysis, the Wilcoxon test was applied (Table 2). The column “V > v %” indicates the effectiveness of particular zones of the Sequential Biofiltration System in the cleaning of sewage; it gives the percentage of measurements in which the concentrations of phosphorus compounds in wastewater at the outflow from the SBS (expressed as “v”) were lower than those at the inflow to the SBS (expressed as “V”), irrespective of their difference (Table 2). The system is more effective at lower concentrations of phosphorus (TP and SRP) flowing through particular beds of the SBS relative to the effluent flowing into the SBS; the highest percentage values in the columns are marked as “V > v %”. Our analysis indicates that TP content falls significantly (p = 0.008) after passing through W5 (the *Glycera maxima* bed) suggesting that the SBS has good effectiveness in cleaning wastewater (Table 2). While TP concentration was found to be lower at W5 than W1 in 69 out of 100 comparisons (69%), it was found to be lower at W8 than W1 in 83% of cases. However, for SRP, a significantly lower concentration was observed at W5 than W1 in 72% of cases, and was lower at W8 than W1 in 75% of cases (Table 2).

**Table 2:** The effectiveness of wastewater TP and SRP concentration reduction in particular zones of the Sequential Biofiltration System (W2–W8) in relation to the concentrations at station W1 (outflow from the WWTP).

	Pair of variables	N	Wilcoxon *p*	Percent V > v
TP	W1 vs. W2	36	0.414	61
	W1 vs. W3	36	0.225	54
	W1 vs. W4	36	0.120	56
	W1 vs. W5	36	0.008 *	69
	W1 vs. W6	36	0.008 *	69
	W1 vs. W7	36	0.001 *	78
	W1 vs. W8	36	0.000 *	**83**
SRP	W1 vs. W2	36	0.387	53
	W1 vs. W3	36	0.489	42
	W1 vs. W4	36	0.162	58
	W1 vs. W5	36	0.002*	72
	W1 vs. W6	36	0.002*	75
	W1 vs. W7	36	0.009*	69
	W1 vs. W8	36	0.000*	**75**

Wilcoxon test (*significance at p < 0.05). The column “V > v %” indicates the percentage of mesurements in which the second variable was lower than the first one. Thirty-six samples were taken from each of eight stations (total number of analysed samples = 288).

### Effectiveness of nitrogen reduction

The biogeochemical cycle of nitrogen is a complex one, with multiple abiotic and biotic transformations involving seven valence states ( + 5 to −3) and a variety of inorganic and organic forms that are crucial for biological life^45^. The most important inorganic forms of nitrogen in wetlands are ammonium (NH_4_^+^), nitrite (NO_2_^−^) and nitrate (NO_3_^−^), and gaseous nitrogen in the form of dinitrogen (N_2_), nitrous oxide (N_2_O), nitric oxide (NO_2_ and N_2_O_4_) and ammonia (NH_3_)^28, 45^.

The N cycle in biofiltration systems is fundamentally different from the soil phosphorus cycle. The retention and removal of nitrogen during wastewater treatment in constructed wetlands (CWs) is dependent on various processes occurring in the system, such as nitrification, denitrification, NH3 volatilization, nitrogen fixation, plant and microbial uptake, ammonification (mineralization), nitrate reduction to ammonium (nitrate-ammonification), anaerobic ammonia oxidation (anammox), fragmentation, sorption, desorption, burial and leaching. However, most processes just convert nitrogen to its various forms, and few processes ultimately remove total nitrogen from wastewater^45^.

In aerobic conditions, the constructed wetlands effectively remove ammonia-N across nitrification process based on the following transformations: ammonia-N (NH_3_^+^- N/NH_4_^+^ -N) → nitrite- N (NO_2−_ -N) → nitrate-N (NO_3_-N), but very little denitrification takes place in these conditions. However, the anaerobic conditions of constructed wetlands are good conditions for denitrification: nitrate-N → nitrite-N → gaseous N_2_, N_2_O, but the ability of these systems to nitrify ammonia is very limited. Therefore, effective removal of total nitrogen is difficult to achieve in constructed wetlands as they cannot provide both aerobic and anaerobic conditions at the same time^45, 47^. Therefore, it is important to combine various types of constructed wetlands and create zones with aerobic and anaerobic conditions to optimise the purification processes and exploit the specific advantages of the individual parts of the CW.

**Figure 4:**
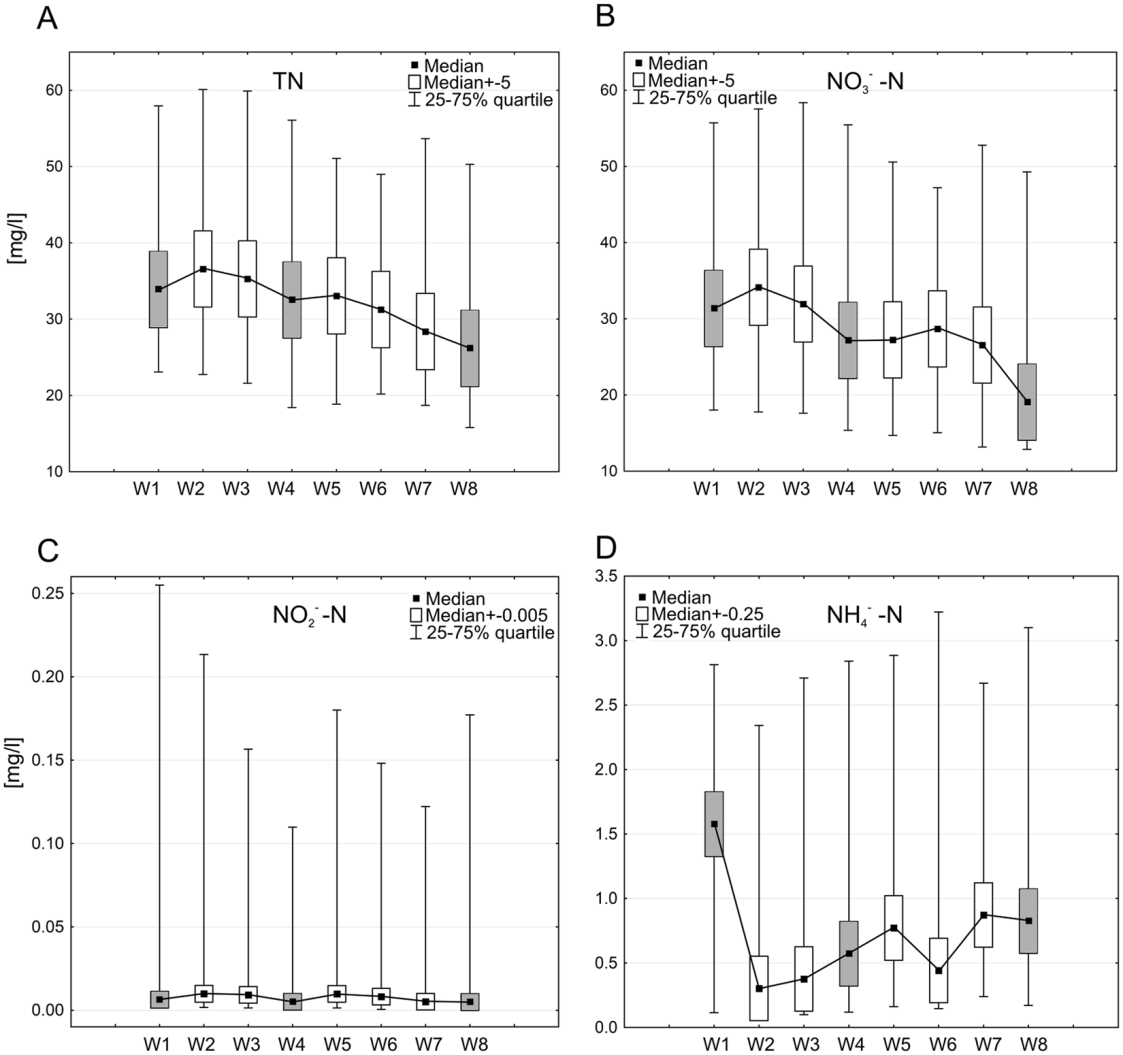
Concentrations of TN (**A**), NO_3_^−^-N (**B**), NO_2_^−^-N (**C**), NH_4_^+^-N (**D**) in wastewater outflowing from the WWTP (W1) and inflowing to individual geochemical (W2–W4) and macrophyte beds (W5–W8) of the Sequential Biofiltration System (SBS). Shaded boxes represent parameters of wastewater at: W1–inflow into the SBS, W4–the point below the geochemical barrier (below the filtration beds), W8–the point below the macrophyte plant barrier. Thirty-six samples were taken from each of eight stations, total number of analysed samples = 288.

Our findings show that concentrations of TN and NO_3_^−^N decreased gradually from inflow to the SBS (W1), across the filtration beds (W2–W4) and via the biologically active beds with macrophytes (W5–W8), to the outflow from the wetland (Fig. 4 and Table 3). Mean concentrations significantly (p = 0.000) decreased from 40.6 to 34.7 mg TN dm^−3^ (mean 15%, maximum 97%) and from 36.5 to 30.2 mg dm^−3^ NO_3_^—^N (mean 17%, maximum 98%), respectively (Table 3). As in the case of TP and SRP, the most significant (p < 0.05) reduction of nitrogen concentration was observed in biologically active beds with macrophytes, particularly below the bed with *Phragmites australis* (W8): the final station located in the outflow from the SBS. The appropriate choice of carbon substrate in the denitrication area in the SBS (W2–W4) results in the removal of all studied nitrogen compounds, despite fluctuating temperatures, higher then optimal pH and nearly anaerobic conditions: The removal of various forms of nitrogen from sewage using this combination of simultaneous nitrification and denitrification results in significantly improved wastewater quality^52, 53^. Although the NH_4_^+^-N concentrations were found to vary widely (Fig. 4), they were found to follow a general trend across the SBS from W1 (3.66 mg dm^−3^), via W5 (4.86 mg dm^−3^), the bed with *Glyceria maxima*, to W8 (4.2 mg dm^−3^), the final biologically-active bed with *Phragmites australis*. The mean increase of NH_4_^+^-N concentration across the SBS amounted to 15%, indicating the presence of the ammonification processes (Fig. 4 and Table 3).

**Table 3:** The comparisons of TN, NO_3_^−^N, NO_2_^−^N, NH_4_^+^-N concentrations in wastewater for all eight monitoring stations (W1–sewage outflow from the WWTP; W2–W8–sewage after treatment in particular zones of the Sequential Biofiltration System).

Parameter	Station	Median	Mean concentration [mg dm^−3^]	N	S.D.
**TN**, ***p*** ** = 0**.000*	W1	33.9	40.6	36	20.9
	W2	36.6	40.5	36	20.3
	W3	35.3	40.9	36	22.6
	W4	32.5	38.9	36	26.0
	W5	33.1	38.4	36	25.9
	W6	31.3	36.7	36	22.1
	W7	28.4	35.1	36	21.7
	W8	26.2	34.7	36	27.1
**% Reduction of TN between W1**–**W8**		**Mean**	**15**		
		**Max**.	**97**		
		Min.	−88		
**NO**_**3**_^**−**^-**N**, ***p*** ** = 0**.000*	W1	31.3	36.5	36	22.9
	W2	34.1	36.7	36	22.8
	W3	31.9	35.9	36	22.5
	W4	27.1	33.7	36	24.6
	W5	27.2	33.2	36	24.2
	W6	28.7	32.7	36	23.4
	W7	26.6	31.3	36	23.2
	W8	19.0	30.2	36	24.1
**% Reduction of NO**_**3**_^**−**^-**N between W1–W8**		**Mean**	**17**		
		**Max**.	**98**		
		Min.	−70		
**NO**_**2**_^**−**^-**N**, *p* = 0.419	W1	0.00629	0.430	36	1.038
	W2	0.00985	0.431	36	1.013
	W3	0.00918	0.441	36	1.047
	W4	0.00503	0.388	36	0.978
	W5	0.00977	0.380	36	0.894
	W6	0.00815	0.368	36	0.862
	W7	0.00512	0.347	36	0.832
	W8	0.00488	0.298	36	0.746
**% Reduction of NO**_**2**_^**−**^-**N between W1–W8**		**Mean**	**31**		
		**Max**.	**100**		
		**Min**.	−933		
**NH**_**4**_^**+**^ **- N**, *p* = 0.341	W1	1.576	3.66	36	6.18
	W2	0.303	3,45	36	6,56
	W3	0.376	4,59	36	8,93
	W4	0.572	4,82	36	9,53
	W5	0.772	4,86	36	9,89
	W6	0.442	3,63	36	6,09
	W7	0.872	3,46	36	5,76
	W8	0.825	4,20	36	9,22
**% Reduction of NH**_**4**_^**+**^ **- N between W1–W8**		**Mean**	**−15**		
		**Max**.	**99**		
		Min.	−3998		

Friedman ANOVA test (*significance at p < 0.05). Percentage reduction of TN, NO_3_^−^N, NO_2_^−^N^,^ NH_4_^+^-N concentrations between stations W1 and W8 during the study period. A negative vale in % Reduction indicates concentration increased in outflowing wastewater. Thirty-six samples were taken from each of eight stations (total number of analysed samples = 288).

Additionally, the analysis of nitrogen compounds present in particular beds shows that both TN and NO_3_^−^N concentrations decrease significantly (*p < 0.05) from station W4 (below the sawdust bed) compared to the values at W1 (Table 4). According to the “V > v %” values, TN concentrations in effluent from the SBS (W8) were significantly lower than effluent from the WWTP (W1) in 69% of examined cases. In the case of NO_3_^−^N, the concentration at W8 was significantly lower than at W1 in 72% of cases, indicating that the SBS has high nutrient removal efficiency (Table 4).

**Table 4:** Effectiveness of the reduction of TN, NO_3_^−^N, NO_2_^−^N, NH_4_^+^-N concentrations in sewage in particular zones of the Sequential Biofiltration System (W2–W8) in relation to the concentrations at station W1 (outflow from STP).

	A pair of variables	N	Wilcoxon *p*	Percent, V > v
TN	W1 vs. W2	36	0.322	58
	W1 vs. W3	36	0.157	61
	W1 vs. W4	36	0.015*	72
	W1 vs. W5	36	0.009*	75
	W1 vs. W6	36	0.009*	78
	W1 vs. W7	36	0.002*	75
	**W1 vs**. **W8**	36	0.002*	**69**
NO_3_^–^-N	W1 vs. W2	36	0.460	56
	W1 vs. W3	36	0.136	64
	W1 vs. W4	36	0.011*	72
	W1 vs. W5	36	0.010*	72
	W1 vs. W6	36	0.009*	81
	W1 vs. W7	36	0.003*	78
	**W1 vs**. **W8**	36	0.002*	**72**
NO_2_^–^-N	W1 vs. W2	36	0.572	47
	W1 vs. W3	35	0.101	46
	W1 vs. W4	36	0.561	50
	W1 vs. W5	36	0.875	53
	W1 vs. W6	36	0.370	58
	W1 vs. W7	36	0.120	64
	**W1 vs**. **W8**	36	0.153	**61**
NH_4_^+^–N	W1 vs. W2	36	0.405	53
	W1 vs. W3	36	0.582	44
	W1 vs. W4	36	0.432	47
	W1 vs. W5	36	0.209	36
	W1 vs. W6	36	0.988	47
	W1 vs. W7	36	0.499	56
	**W1 vs**. **W8**	36	0.850	**44**

Wilcoxon test (*significance at p < 0.05). The column “V > v %” indicates the percentage of mesurments in which the second variable was lower than the first. Thirty-six samples were taken from each of eight stations (total number of analysed samples = 288).

### Effectiveness of PCB EQ removal

The increased global usage of water has led to amplified apprehension about the quality of wastewater treated and discharged into the water bodies through municipal WWTPs^12^. Thus far, the analysis of treated wastewater quality has been restricted to monitoring traditional parameters regulated by the European Urban Wastewater Directive such as biochemical oxygen demand, chemical oxygen demand, nitrates, phosphates and Total Suspended Solids, which are easily and inexpensively measured (91/271/EEC)^14^. In contrast, toxic organic compounds, such as PCBs have not been monitored in such a routine way. What is more, the plethora of data demonstrates that WWTPs are not efficient at removing PCBs^11–15, 17^. Consequently, these compounds are detected not only in raw and treated wastewater, but also in rivers^7, 8, 59^, thus posing a threat to the whole river ecosystem.

In view of the above, a promising solution for alleviating the pollution of river by PCBs and other organic compounds is through the use of tertiary treatment to complement the traditional method employed in the WWTPs. One of such tertiary purification method is the use of SBS comprising a set of filtration (mineral and organic) and wetland beds which enables the removal of not only nutrients but also organic compounds by a range of physicochemical and biogeochemical processes.

**Figure 5:**
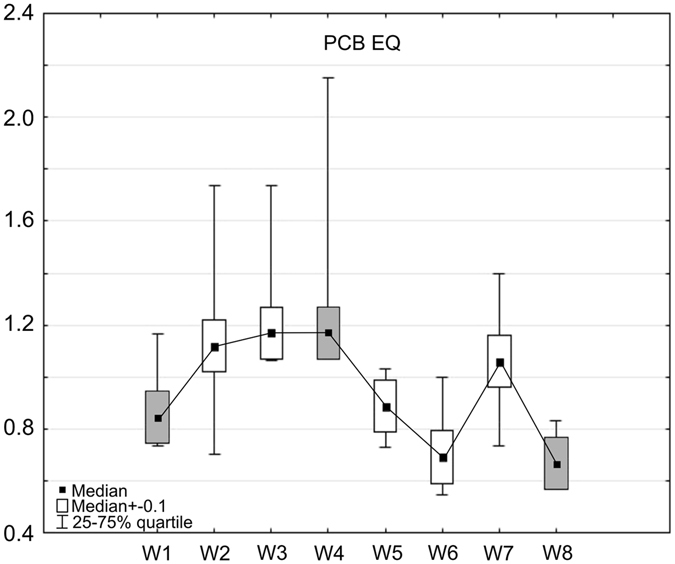
Concentrations of PCB EQ [µg dm^−3^] in wastewater effluent from WWTP (W1) and when entering particular geochemical (W2–W4) and macrophyte beds (W5–W8) of the Sequential Biofiltration System. Shaded boxes represent parameters of wastewater at: W1–inflow into the SBS, W4–at a point below the geochemical barrier (below filtration beds), W8–at a point below the macrophyte barrier. Five samples were taken at W1–W4 and four samples at W5–W8 stations, total number of analysed samples = 36).

Our results showed that the entire SBS has a mean removal efficiency, in terms of PCB EQ, of about 21% (Table 5); however, particular SBS beds demonstrated different rates of PCB EQ removal. The mineral and organic beds in the present study (W1–W4) demonstrated an increase in the concentrations of PCB EQ ranging from 0.85 µg dm^−3^ (W1) up to 1.17 µg dm^−3^ (W3 and W4) (Fig. 5 and Table 5). The wetland part of the SBS showed the opposite influence, with the average PCB EQ concentration decreasing from 1.17 µg dm^−3^ at W4 to 0.67 µg dm^−3^ at W8 (Table 5); however, a slight increase of up to 1.06 µg dm^−3^ was observed at W7: a bed planted with *Typha latifolia*. The greatest decline in PCB EQ concentration was observed between sampling point W7 (wastewater flowing from the *Typha latifolia* bed) and W8 (wastewater flowing from the *Phragmites australis* bed): a 38% reduction of the analysed compounds (Fig. 5). The greatest efficiency in this regard (43%) was obtained using beds with macrophytes (W5–W8), while a 38% increase in the PCB EQ concentration was found for the filtration beds (W2–W4) (Table 5).

**Table 5:** Median concentrations of PCB EQ and its removal efficiency by individual geochemical beds (W1–W4), and macrophyte beds (W5–W8), and across the whole Sequential Biofiltration System (W1–W8).

PCB EQ concentrations [µg dm^−3^	
Median PCB EQ concentration at W1	0.85
Median PCB EQ concentration at W4	1.17
Median PCB EQ concentration at W8	0.67
**PCB EQ removal effectiveness (%)**	
Removal of PCB EQ by mineral and organic beds (W1–W4)	−38
Removal of PCB EQ by wetland with macrophytes (W5–W8)	43
Removal of PCB EQ by whole SBS (W1–W8)	21

A negative value indicates increased concentration in the outflow. Five samples were taken at W1–W4 and four samples at W5–W8 stations, total number of analysed samples = 36.

The obtained results demonstrated that the wetland zone was well suited to PCB EQ removal. This can be related to the phytodegradation properties of selected macrophytes^54^. Chu *et al*.^55^ found *P*. *australis* possess enzymes degrading PCB with up to three chlorine atoms, while higher chlorinated congeners were not transformed. Toro-Valez *et al*.^56^ report the removal of Bisphenol A (mean value 70.2%) and nonylphenols (mean value 52.1%) by wetlands planted with *P*. *australis*. Also, microorganisms play a vital role in the degradation of PCBs in constructed wetlands^57^. Reddy and D’Angelo^58^ have discussed the pathways and indicators for toxic organic compound removal in constructed wetlands. They hypothesize that the removal of organic compounds is largely a microbially mediated process, and can be subdivided into aerobic and anaerobic microbial degradation processes. In the case of PCBs, the main factor limiting their microbial degradation is their low availability resulting from binding to the wetland sediment/soil matrix. The compounds become more soluble and thus more available after some initial reductive dechlorination that occurs under anaerobic conditions. This step leads to the production of less chlorinated congeners, which can then be used in aerobic transformations. The input of oxygen by macrophytes in turn creates aerobic conditions and thus promotes the microbial aerobic degradation of PCBs. Consequently, the mutual cooperation of wetland macrophytes and microbiota (rhizodegradation) enhances PCB removal, improving the quality of outflowing wastewater and protecting river ecosystems.

### Removed of phosphorus, nitrogen and PCB load

The average daily loads of phosphorus and nitrogen compounds and PCB EQ load of the WWTP outlet (W1) and in particular zones (W2–W8) of the SBS are given in Table 6. Total outflow of contaminant load from WWTP per year (at the inlet to the SBS) amounted to 139.2 kg TP, 143.5 kg SRP, 1096.7 kg TN, 949.9 kg NO_3_^−^N, 13.4 kg NO_2_^−^N and 133.4 kg NH_4_^+^-N and 27.9 g PCB EQ (Table 6).

**Table 6:** Average daily P, N and PCB loads in sewage in outlet (W1) of WWTP and transported via particular zones (W2–W8) of the hybrid Sequential Biofiltration System (SBS).

Station	Average daily load of nutrient						Average daily load of PCB EQ
	TP	SRP	TN	NO_3_^−^–N	NO_2_^−^–N	NH_4_^+^–N	
	kg day^−1^	kg day^−1^	kg day^−1^	kg day^−1^	kg day^−1^	kg day^−1^	mg day^−1^
W1	0.38	0.39	3.00	2.60	0.04	0.37	76.56
W2	0.39	0.38	2.99	2.60	0.04	0.35	92.67
W3	0.38	0.40	3.00	2.54	0.04	0.42	113.83
W4	0.37	0.36	2.86	2.39	0.03	0.44	124.52
W5	0.35	0.35	2.79	2.33	0.03	0.43	50.11
W6	0.36	0.34	2.66	2.26	0.03	0.37	43.12
W7	0.35	0.32	2.57	2.19	0.03	0.35	59.52
W8	0.31	0.29	2.48	2.11	0.02	0.34	39.26
**Average daily removed load of nutrient** (kg day^−1^) **and PCB EQ** (mg day^−1^) in SBS via station W1–W8	**0.07**	**0.10**	**0.52**	**0.49**	**0.01**	**0.02**	**37.30**
**Total outflow of nutrient** (kg yr^−1^) **and PCB EQ** (g yr^−1^) **load from the WWTP** (without SBS)	139.2	143.5	1096.7	949.9	13.4	133.4	27.9
**Percentage removed load of nutrient and PCB EQ in SBS** (%)	18	26	17	19	33	7	49
**Total removed load (TRL) of nutrient** (kg yr^−1^) **and PCB EQ** (g yr^−1^) **in whole SBS**	**25.3**	**37.1**	**191.3**	**178.0**	**4.5**	**8.8**	**13.6**
**Total removed load (TRL) of nutrient** (kg yr^−1^) **and PCB EQ** (g yr^−1^) **calculated per m** ^**2**^ **of SBS**	**0.415**	**0.607**	**3.136**	**2.919**	**0.073**	**0.144**	**0.223**

Total outflow of nutrient and PCB EQ load from the WWTP and load of these contaminants in SBS removed during the one-year study period.

The hybrid SBS removed 0.07 kg TP, 0.1 kg SRP, and 0.52 kg TN, 0.49 kg NO_3_^−^N, 0.01 kg NO_2_^−^N, 0.02 kg NH_4_^+^ - N and also 37.3 mg of PCB EQ from wastewater on a daily basis (Table 6). Total annual contaminant load removal: 25.3 kg TP (18%), 37.1 kg SRP (26%), 191.3 kg TN (17%), 178 kg NO_3_^−^N (19%), 4.5 kg NO_2_^−^N (33%), 8.8 kg NH_4_^+^ - N (7%) and also 13.6 g PCB EQ (49%) (Table 6). Hence, the SBS removed a significant load of phosphorus (0.415 kg TP; 0.607 kg SRP), nitrogen (3.136 kg TN; 2.919 kg NO_3_^−^N; 0.073 kg NO_2_^−^N; 0.144 kg NH_4_^+^-N), and PCB EQ (0.223 g) per square meter (Table 6).

Vymazal^45^ reported TP load removal ranging between 0.045 and 0.075 kg m^−2^ yr^−1^ and TN load removal ranging between 0.25 and 0.63 kg m^−2^ yr^−1^ in constructed wetlands using sorption substrates depending on CW system type, water flow regime (horizontal or vertical flow) and inflow nutrient loading. Of course, a greater area of wetlands was associated with a greater nutrient holding capacity^35^. In addition, harvesting the aboveground biomass of emergent vegetation could increase nutrient removal by around 10–20 g P m^−2^ yr^−1^ and 100 –200 g N m^−2^ yr^−1^ ^45^. Our study returned greater load removal values for both phosphorus and nitrogen (Table 6) than noted by Vymazal^45^, perhaps because the combination of a geochemical barrier with sequential filtration beds and a biological active barrier with macrophyte beds is much more effective than simple wetland. Forbes *et al*.^60^ report that a constructed wetland system in agricultural land in Northern Ireland treating wastewater from a farm with 170 cows gave an mean accumulation of P of 26 kg per year, which is comparable to our value of 25.3 kg TP.

The significant removal of nutrients, especially TP and SRP, between W1 and W8 may be attributed to the binding of calcium compounds and accumulation in PAO (Phosphate-Accumulating Organism) bacteria. These bacteria are able to accumulate polyphosphates as metaphosphates and polyphosphates in amounts of up to 25% of dry weight. This increased accumulation of polyphosphates in PAO microbial cells could be induced in response to their movement from an environment with a relatively low content of phosphorus compounds to an environment very rich in them^61^. This mechanism is characteristic of microorganisms inhabiting surface waters and wastewater. The use of carbon beds in biofilters, as in our SBS, may provide a good substrate for the settlement of bacteria which help reduce nitrogen in denitrification zones. Denitrifying bacteria, such as those activated in the carbon and sawdust bed in the hybrid SBS (W3–W4), remove phosphate from both water and wastewater. Increasing the activity of Phosphate-Accumulating Organisms is a cheap and efficient method of removing biological phosphorus. For this reason, the use of associated geochemical and biological methods represents the most effective solution for deliberate and targeted modification of the circulation of phosphorus and nitrogen in the environment.

Concerning the total removal of PCB EQ load, the obtained results showed that SBS removed 13.6 g yr^−1^ of PCB EQ, constituting 49% of its total load entering the constructed system (Table 6). Hence, the hybrid SBS removed 0.223 g yr^−1^of PCB EQ load per square mater. Of all the examined compounds, the system was most effective at removing PCB EQ, indicating that the SBS was suitable for removing not only nutrients, but more importantly, organic compounds prioritized as harmful for the water ecosystems (EQS Directive, 2013/39/EU).

## Materials and Methods

### Sequential Biofiltration System - experiment design

The experiment was conducted in central Poland, which has a transitional climate with an annual average air temperature of 8 °C. The average temperatures are between 16 °C and 20 °C in the summer, and between −6 °C and 0 °C in the winter. The hottest month is July and the coldest January. The average precipitation in central Poland is approx. 600 mm per year. The distribution of precipitation during the year is uneven, with 75% of the annual rainfall in spring and summer.

The hybrid Sequential Biofiltration System (SBS) was designed and constructed at the Wastewater Treatment Plant in the town of Rozprza (central Poland, 51°18′08″N, 19°38′45″E). The horizontal-flow SBS system was constructed in a ditch carrying discharged treated wastewater from the WWTP to the Dąbrówka River (a tributary of the Pilica River): the discharged sewage requires thorough cleaning. The Rozprza Wastewater Treatment Plant is a small-sized plant working on 500 population equivalent (p.e.), whose average daily outflow of sewage is 107 m^3^ across a multi-year period, and 79.1 m^3^ in the study period. The SBS was built on a gradient to ensure that the effluent would flow through each wetland bed under gravity. The total area of the SBS is 61 m^2^ and includes seven beds: three filtration beds of the total area 21 m^2^ and four biologically active beds with macrophytes of a total area of 40 m^2^ (Fig. 1). The filtration zone includes the following beds: a limestone bed with an area of 7 m^2^ (2 m Width; 3.5 m Length; 0.8 m Depth) with the limestone ranging from 4 to 7 cm diameter, a coal bed with an area of 7 m^2^ (2 m W; 3.5 m L; 0.8 m D) with the coal ranging from 4 to 7 cm diameter, and a sawdust bed with an area of 7 m^2^ (2 m W; 3.5 m L; 0.8 m D). The sawdust bed consisted of sawdust was placed in raschel bags with a mesh size of 1 cm. The substrate in these filtration beds was sealed and isolated using black foil with a thickness of 1.5 mm. In the ditch, below the three filtration beds, four biologically-active beds with transplanted young aquatic macrophytes were constructed: *Glyceria maxima* Hartm., *Acorus calamus* L., *Typha latifolia* L., *Phragmites australis* (Cav.) Trin. ex Steud (Fig. 1). The surface area of each of the four constructed wetland macrophyte beds was 10 m^2^ (2.0 m W; 5.0 m L) and the total area of the biologically active zone was 40 m^2^. The plants were planted at a distance of 20 cm apart, giving a density of 36 specimens m^2^, in a geotextile mat mounted in the ditch. The mat acted as the frame for the young plants, helped their rooting and secured the plants from being flushed out by the flowing sewage.

### Sampling

The physical and chemical parameters of wastewater collected from the SBS were measured at the following eight stations (Fig. 1): W1–outflow from WWTP and inflow to SBS, W2–station located behind the limestone bed, W3–station located behind the coal bed, W4–station behind the sawdust bed, W5–station behind the *Glyceria maxima* bed, W6–behind the *Acorus calamus* bed, W7–behind the *Typha latifolia* bed, W8–behind the *Phragmites australis* bed.

Samples of wastewater were taken at one-week intervals for the analysis of nutrient concentrations (36 sampling periods at each station, 288 samples for all stations) between May 2012 and April 2013. Each time, two samples of 1 dm^3^ were collected. From each monitoring station, one filtered sample was taken to analyse the concentrations of Total Suspended Solids (TSS) and dissolved forms of nutrients, and a second unfiltered sample was taken to analyse the total forms of nutrients. Subsequently, two subsamples of 0.1 dm^3^ were taken from the latter, one to analyse the dissolved forms of the nutrients and the second to analyse the total forms of the nutrients. The samples were transported to a laboratory in a refrigerator at a temperature of 4 °C.

Wastewater samples for PCB analysis were collected at monthly intervals between June and October 2012. The 1 dm^3^ samples were collected to glass vials and transported in the dark at 4 °C to the laboratory for further PCB analysis.

### Analysis of physical parameters and nutrients concentration in wastewater

The physical parameters, such as temperature, oxygen concentration, pH and conductivity, were measured by a YSI Professional Plus multiparameter meter. Each 1 dm^3^ wastewater sample was filtered through Whatman GF/F 0.45 m filters, which were then dried at 105^◦^C and weighed on a laboratory scale to estimate TSS content. The concentration of TSS was determined by finding the difference in weight of the filter before filtration and after drying at 105^◦^C.

Wastewater samples for soluble forms of nutrients, e.g. Soluble Reactive Phosphorus (SRP), NO_3_^−^-N, NO_2_^−^-N, NH_4_^+^-N, were filtered through Whatman GF/C 0.45 m filters and analysed with the Ion Chromatography System (DIONEX, ICS 1000). Total phosphorus (TP) in unfiltered wastewater samples as processed with the oxidizing decomposition reagent Oxisolv (Merck) and the Merck MV500 Microwave Digestion System, with the total amount determined by the ascorbic acid method^62^. Total nitrogen (TN) was analysed using the persulphate digestion method^63^. The daily volumes of wastewater outflow from the WWTP and the concentrations of nutrients in wastewater were then used to calculate the annual daily load.

### Analysis of PCB in wastewater

PCBs contents were analysed using Enzyme Linked Immunosorbent Assay (ELISA)–PCB RaPID Assay, according to Wyrwicka *et al*.^64^. Aliquots (0.0002 dm^3^) of calibration-standard PCB (0, 2.25, 1, 5 µg dm^−^^3^), the wastewater sample, and a positive control solution (6 ppm) were added to test tubes together with aliquots of enzyme conjugate (0.00025 dm^3^). An aliquot (0.0005 dm^3^) of antibody, coupled with magnetic particles in buffered saline containing preservative and stabilizers, was the added, thoroughly mixed and incubated at room temperature for 15 minutes using a RaPID Magnetic Separator. After incubation, the contents of each vial were decanted to a waste container to remove the solution containing any unbound reagents. The vials were then washed twice with washing solution (0.001 dm^3^ per vial). Following washing, an aliquot (0.0005 dm^3^) of colour solution containing hydrogen peroxide and 3,3′,5,5′-tetramethylobenzidine in an organic base was added to each vial, shaken and incubated for 20 minutes. At the end of the incubation period, an aliquot (0.0005 dm^3^) of stopping solution, containing 2 M sulphuric acid, was added to each vial. The absorbance of the liquid in each vial was measured at 450 nm using a SDI Differential Spectrophotometer. The concentrations of the samples were determined using a standard curve and presented as ELISA-equivalency (ELISA-EQ) values.

For the correct performance of the PCB EQ analysis, the QC/QA method was applied. Each analytical batch contained a sample blank, a control sample of known concentration (3 µg dm^−3^, as Aroclor 1254), calibration standards and samples. The precision was verified by duplicate analyses and the test reproducibility was measured using the coefficient of variation (CVs). The CVs should be lower than 10% for standard duplicates. If the CVs exceeded the above values, the whole procedure was repeated in order to achieve good quality of the obtained results. The minimum method detection limit was 0.20 µg dm^−3^.

### Statistical data analysis

Data analysis was conducted using Microsoft Excel 2010 and STATISTICA 10 (StatSoft, 2011). The analysed data was collected by repeated measurements in the form of a matrix with *n* rows and *k* columns. Rows correspond to objects and columns to measurements, giving *n* objects and *k* groups of dependent measurements. The number (n) of objects is not large enough to verify whether the data inside the columns is normally distributed. Therefore, nonparametric tests were chosen for further analysis.

The Friedman ANOVA test was used if *k* > 2 and the Wilcoxon test, when *k* = 2. As with all nonparametric ones, the tests are based on ranks. In case of Friedman, the data ranks are established separately for each of *n* rows. The significance level *p* is found as a function of *k* sums computed separately for each column.

The Wilcoxon test requires a calculation of the differences between paired measurements in dependent groups 1 and 2, then ranking them by absolute values. The rows with differences equal to zero are omitted. Next, all ranks associated with positive differences are summarized. This sum represents a statistic for calculating the significance level *p*. The Wilcoxon test was used to compare the nutrient concentrations in sewage from the outlet of the WWTP at station W1 with those in sewage after cleaning in particular beds of the SBS at stations W2–W8 (*significance at p ≤ 0.05). The physical and chemical parameters of the wastewater at the inflow and outflow points of the SBS, together with hydrological data, was used to evaluate the efficiency of the biofiltering system. Reduction of nutrient concentration in the SBS from stations W1 to W8 was calculated according to the basic equation (Eq. 1) (adapted from O’Neill *et al*.)^35^:

1 $${{\rm{Cw}}}_{1}-{{\rm{Cw}}}_{8}={\rm{Reduction}}$$

Where: C_W1_ is the concentration of nutrients/PCBs in the wastewater at inflow to the SBS (first monitoring station W1); C_W8_ is a concentration of contaminant in the wastewater at the last monitoring station (W8) of the SBS.

The percentage reduction of concentration was calculated according to the equation (Eq. 2):

2 $$ \% \,{\rm{Reduction}}=[100-({{\rm{C}}}_{{\rm{W}}8}\ast 100)/{{\rm{C}}}_{{\rm{W}}1}]$$

The average daily load of nutrients (kg day^−1^) and PCB EQ (mg day^−1^) was calculated by multiplying the daily outflow of wastewater from the sewage treatment plant (Q, expressed in m^3^ day^−1^) by the concentration of nutrients (expressed in mg dm^−3^) and PCB EQ (expressed in µg dm^−3^) in wastewater.

Total removed load (TRL), expressed in kg yr^−1^, is the total load of nutrient compounds removed by the SBS system. TRL was calculated according to the equation (Eq. 3):

3 $${\rm{TRL}}=({{\rm{Lw}}}_{1}-{{\rm{Lw}}}_{8})\ast 365$$

Where L_W1_ is the average daily load of nutrient in wastewater outflowing from WWTP and inflowing to the Sequential Biofiltration System (W1); L_W8_ is average daily load of nutrient in treated sewage outflowing from the last bed (W8) of the SBS; assuming 365 days in a year.

## Conclusions

The hybrid Sequential Biofiltration System (SBS) proved an effective method of ecohydrological biotechnology for treatment of wastewater from WWTPs. The SBS removed the following contaminant loads: 25.3 kg TP (18%), 37.1 kg SRP (26%), 191.3 kg TN (17%), 178 kg NO_3_^−^N (19%), 4.5 kg NO_2_^−^N (33%), 8.8 kg NH_4_^+^-N (7%) and also 13.6 g PCB EQ (49%) per year. The greatest reduction of concentration was observed in the biologically-active barrier, i.e. beds with macrophytes, wherein 83% of TP, 75% of SRP, 69% of TN, 72% of NO_3_^—^N, and 43% of PCB EQ concentrations were lower at the outlet from the SBS than the inlet. The low-cost Sequential Biofiltration Systems may be used in small WWTPs as an additional treatment step and an alternative ecohydrological biotechnology for reducing point source pollution and improving water quality in river catchments.
